# Abdominal Pain in the Elderly Patient: Point-of-care Ultrasound Diagnosis of Small Bowel Obstruction

**DOI:** 10.5811/cpcem.2020.11.50029

**Published:** 2021-01-22

**Authors:** Ahmad Hussein, Alexander Arena, Connie Yu, Angela Cirilli, Ellen Kurkowski

**Affiliations:** St. John’s Riverside Hospital, Department of Emergency Medicine, Yonkers, New York

**Keywords:** Abdominal pain, small bowel obstruction, point-of-care ultrasound

## Abstract

**Case Presentation:**

A 67-year-old female presented to the emergency department (ED) complaining of generalized abdominal pain, nausea, and vomiting. Point-of-care ultrasound (POCUS) demonstrated dilated bowel loops measuring up to 4.1 centimeters and localized free fluid, consistent with a small bowel obstruction (SBO). A nasogastric tube was placed without complications. The patient was admitted to the hospital and conservatively managed with an uncomplicated course.

**Discussion:**

In elderly patients with abdominal pain, POCUS is an excellent initial imaging modality to assist emergency physicians in rapid and accurate diagnosis of a variety of pathologies to expedite management. Point-of-care ultrasound can be used to rule out and evaluate for conditions encountered in emergency medicine, including acute cholecystitis, renal colic, abdominal aortic aneurysm, and intraperitoneal free fluid. As demonstrated in our case presentation, POCUS had an integral role in the early diagnosis and management of a SBO.

## CASE PRESENTATION

**Image 1:**
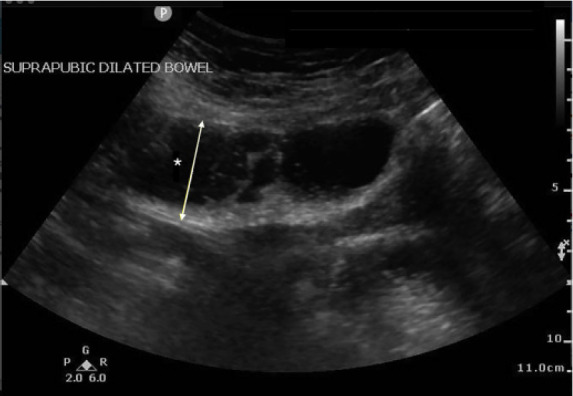
Point-of-care ultrasound demonstrating long-axis view of dilated, fluid-filled loops of bowel (*) measuring 4.1 centimeters from outer bowel wall to outer wall, consistent with small bowel obstruction.

**Image 2:**
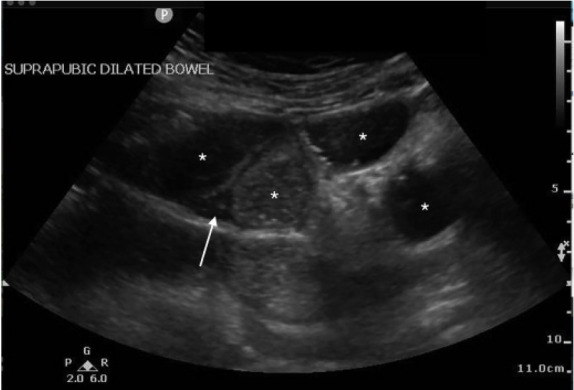
Point-of-care ultrasound demonstrating short-axis view of dilated loops of bowel (*) with localized surrounding free fluid (white arrow) consistent with small bowel obstruction.

A 67-year-old female with a history of hypertension, diabetes, and exploratory laparotomy presented with abdominal pain for one day associated with nausea, nonbloody-nonbilious emesis, and normal bowel movements. Abdominal exam revealed a soft, nondistended abdomen with a laparotomy scar and both periumbilical and right lower quadrant tenderness. Point-of-care ultrasound of the abdomen was performed with no acute abnormalities of the gallbladder or kidneys. The “lawn mower” method, scanning systematically across all abdominal quadrants in a horizontal or vertical fashion, demonstrated dilated, small bowel loops containing air and fluid measuring up to 4.1 centimeters (cm) (Image 1) with localized free fluid (Image 2). Computed tomography (CT) of the abdomen confirmed the diagnosis. Following nasogastric tube placement, the patient was admitted with an uncomplicated hospitalization course.

## DISCUSSION

Small bowel obstruction (SBO) is a common emergency department diagnosis estimated to comprise 2% of all patients with abdominal pain, resulting in 300,000 hospitalizations per year with etiologies including adhesions, neoplasms, hernias, and Crohn’s disease.^1^,^2^ Expeditious diagnosis of SBO can prevent potential complications, including bowel ischemia, necrosis, and perforation.^1^,^2^ Studies suggest that point-of-care ultrasound is highly accurate in diagnosing SBO with sensitivity and specificity of 92% and 94–96%, respectively, compared to CT imaging.^1^,^3^ The most sensitive sonographic findings include dilated bowel loops and abnormal peristalsis.^1^,^3^,^4^,^5^ Fluid-filled small bowel loops measuring greater than 2.5 cm is highly indicative of SBO.^4^,^5^ Abnormal peristalsis is manifested by “to-and-fro” or swirling of intraluminal bowel contents.^4^,^5^ More specific sonographic signs include bowel wall edema if plicae circulares project into the bowel lumen (“keyboard sign”); free fluid between adjacent bowel loops; and the identification of a transition point.^4^,^5^ Point-of-care ultrasound is a useful screening tool in the early diagnosis and management of SBO in emergency medicine, where evolving literature has shown reasonable diagnostic accuracy with time and cost-saving implications.

**CPC-EM Capsule**

What do we already know about this clinical entity?

Small bowel obstruction (SBO) is a common presenting diagnosis in the Emergency Department (ED) that requires prompt evaluation and intervention.

What is the major impact of the image(s)?

Point-of-care ultrasound (POCUS) has emerged as a useful, rapid and noninvasive screening tool in the early diagnosis and management of SBO in the ED

How might this improve emergency medicine practice?

Early diagnosis of SBO in the ED allows for improved patient care by initiating the appropriate intervention as well as surgical evaluation.

## Supplementary Information

Video. Point-of-care ultrasound demonstrating “to-and-fro” swirling of bowel intraluminal contents signifying abnormal peristalsis consistent with small bowel obstruction.
